# Comprehensive analysis of the clinical feature, myeloid neoplasm-related gene mutation profiles and T cell diversity acquired pure red cell aplasia

**DOI:** 10.1007/s00277-025-06638-x

**Published:** 2025-09-25

**Authors:** Yuemin Gong, Xingxing Chai, Xiaoqing Liu, Yawen Zhang, Yue Li, Yunlong Li, Jianping Hao, Guangsheng He

**Affiliations:** 1 Department of Hematology, Jiangsu Province Hospital, Key Laboratory of Hematology of Nanjing, Collaborative Innovation Center for Cancer Personalized Medicine, the First Affiliated Hospital with Nanjing Medical University, Nanjing, 210000 China; 2 Department of Hematology, the Second People’s Hospital of Lianyungang, Lianyungang, 230601 China; 3 Department of Hematology, the Second Hospital of Nanjing, Nanjing University of Chinese Medicine, Nanjing, 210037 China; 4 Chongqing Hospital of Jiangsu Province Hospital, the People’s Hospital of Qijiang District Chongqing, Chongqing, 400800 China; 5 Department of Hematology, Xinjiang Hematology Clinical Medical Research Center, Xinjiang Institute of Hematology, First Affiliated Hospital of Xinjiang Medical University, No.137 Liyushan Road, Urumqi , 830054 China

**Keywords:** Gene mutation, Large granular lymphocytic leukemia, Pure red cell aplasia, T-cell receptor rearrangement

## Abstract

**Purpose:**

Acquired pure red cell aplasia (PRCA) is a kind of rare bone marrow failure disease characterized by destruction of erythrocyte by the immune system. However, the diverse etiologies of acquired PRCA make its pathogenesis largely unclear.

**Materials and methods:**

We portrayed the clinicopathologic, mutation and TCR rearrangement profiles of 64 primary PRCA cases and 104 large granular lymphocytic leukemia (LGLL)-associated PRCA, and tried to reveal the association factors of CsA response.

**Results:**

We found that gene mutations were detected in 39.7% of acquired PRCA who were older than 40 years, with *DNMT3A*, *KMT2A* and *TP53* being the top 3 mutation genes. KMT2A mutation was only detected in patients with normal reticulocyte (Ret)%, while IDH1 mutation only occurred in patients with normal CD3 + CD8+/Lym%. For LGLL-associated PRCA patients, TRBV6_TRBJ2 was the most frequent dominant clonotype and the proportion of each dominant clone decreased following the remission of anemia. The response rate of LGLL-associated PRCA to CsA treatment was lower than primary PRCA (56.4% vs. 77.4%). β2-MG dysregulation, MF dysregulation were unfavorable factors for the response to CsA in PRCA patients, while other clinical information, mutated genes, number of mutated genes, mean VAF, number of TCR clones in PRCA patients did not significantly affect the response to CsA.

**Conclusion:**

This study described the clinical features, mutation landscape and TCR rearrangement profile in a relatively larger PRCA cohort, which may contribute to the clear perception of PRCA and the development of more potent treatment approaches.

**Supplementary Information:**

The online version contains supplementary material available at 10.1007/s00277-025-06638-x.

## Introduction

Pure red cell aplasia (PRCA), mostly refers to the acquired disease entities, is a rare bone marrow failure disease characterized by normocytic normochromic anemia, and often manifests as the absent or infrequent erythroblasts in bone marrow [1, 2]. PRCA is generally divided into primary PRCA and secondary PRCA based on whether there is an exact cause [3, 4]. Specially, primary PRCA is an autoimmune disease which is commonly mediated by autoantibodies [5]. Secondary PRCA is triggered by different causes, including systemic lupus erythematosus, rheumatoid arthritis, lymphoproliferative disorders, solid tumors, and viral/bacterial infections, among which the large granular lymphocytic leukemia (LGLL) and thymoma are the top two reasons [2, 6]. PRCA is a highly heterogeneous disease with multifaceted nature, as a consequence, the treatment for this disease is diverse and strictly dependent on the presented clinical scenario, such as cyclosporine A (CsA), corticosteroids (CS), cyclophosphamide (CTX), anti-thymocyte globulin (ATG) and rituximab [6–8]. Means et al. [1] reported that CsA had better therapeutic effect on PRCA than CS, but some studies indicated that CsA and CS produced similar remission rates in primary PRCA patients [6, 9]. The combination of CTX plus steroids seems to be particularly effective in the context of LGLL-associated PRCA, as shown by the prolonged remissions when compared with other treatment modalities (steroids alone, CsA, or methotrexate) [10]. Thus, it is important to recognize the pathogenetic clues because of their diagnostic and therapeutic implications.

PRCA together with aplastic anemia (AA), myelodysplastic syndrome (MDS), and paroxysmal nocturnal hemoglobinuria (PNH) is the main constituent part of bone marrow failure (BMF) syndromes. BMF syndromes could transit from one type to another [11, 12], indicating PRCA may share a common pathophysiology with other BMF syndromes. To this end, the mutational landscape of BMF syndromes including some types of PRCA have been portraited. Specifically, 20 ~ 30% of PRCA cases are reported to carry myeloid clonal hematopoiesis-related gene mutations [13]. These CH-related mutations are sometimes benign, but may still be related with poor overall survival, increased risk of myeloid neoplasms development and all-cause mortality [14]. In addition to the CH-related mutations, Long et al. [15] portraited the mutation profile of 30 patients with acquired PRCA, and found that *BCOR*, *BCORL1*, and *CSMD1* are the most frequently mutated genes (10%), followed by *JAK3* and *RUNX1* (6.7%). Also, the researchers found that the patients with *BCOR* or *BCORL1* mutations had a similar response to immunosuppressive therapy (IST) compared to those without any mutation, but had a better response than patients with other gene mutations. Kawakami et al. [16] recently reported that 43% of PRCA cases carried *STAT3* mutations, and *STAT3* mutations were associated with poor responsiveness to CsA. However, current research data have only been obtained from small sample sizes.

Acquired PRCA is usually considered as an immune-mediated disease with chronic CD8 + T cell expansion. Thus, clinical and subclinical T-cell clones can be detected in a subset of aplastic anemia and MDS regardless of LGLL [16, 17]. Balasubramanian et al. [18] reported the clonal rearrangements of TCR (T cell receptor) gene were positive in all 14 cases with LGLL-associated PRCA, 33% of thymoma-associated and 20% of idiopathic cases. Noticeably, T cell diversity also represents an important metric of the T cell space with its own prognostic significance [19]. However, the specific rearrangement model of TCR gene and its relationship with response to treatment remains largely unknown.

In this study, we described the clinicopathologic, mutation and TCR rearrangement profiles in 168 PRCA patients including 64 primary PRCA cases and 104 LGLL-associated PRCA. Also, we investigated their associations with clinicopathologic features and response to CsA, aiming to expand the knowledge base of PRCA and improve the treatment response.

## Materials and methods

### Patient cohort

A total of 168 patients with acquired PRCA aged ≥18 years (primary PRCA, *n* = 64; LGLL-associated PRCA, *n* = 104) diagnosed in the First Affiliated Hospital of Nanjing Medical University/Jiangsu Provincial People’s Hospital from October 2014 to December 2022 were included in this study. PRCA was diagnosed based on the following criteria: (1) Red cells in PRCA are normochromic and normocytic. The absolute reticulocyte count is always less than 10,000/µL; (2) Bone marrow examination results show absence or near absence of erythroblasts from an otherwise normal marrow. T-LGLL-related PRCA patients met the criteria for T-LGLL with the following aspects: (1) Cytology: Typical large granular lymphocyte morphology in peripheral blood or bone marrow with a sustained increase for over 6 months (> 0.5 × 10^9^/L). (2) Immunophenotype: the abnormal immunophenotype is characterized by a TCR αβ or γδ phenotype; the typical phenotype of CD8 + T-LGLL includes CD3 + CD8 + CD57 + CD16 + CD4-; CD4 + T-LGLL commonly exhibits immunophenotypes such as CD4 + CD57 + CD8- or CD4 + CD8 + dim; TCRαβ + T cells in this context show restricted expression of TRBC1. (3) Evidence of clonality: positive TCR gene rearrangement or confirmation of monoclonal expression via TCRVβ lineage flow cytometry for T-LGLL; *STAT3* gene detection helped exclude reactive amplification. Simultaneous diagnosis of T-LGLL and PRCA indicated T-LGLL-associated PRCA. General clinical information included sex, age, hemoglobin (Hb), reticulocyte percentage (Ret%), red cell distribution width-coefficient of variation (RDW-CV), absolute neutrophil count (ANC), platelet (PLT), ferritin, lactate dehydrogenase (LDH), beta2-microglobulin (β2-MG), mean corpuscular volume (MCV), myelofibrosis (MF), lymphocyte percentage (Lym%), CD3 + CD8+/Lym%, CD3 + CD4+/Lym%, CD3 + CD57+/Lym%, treatment response to cyclosporine (CsA) were collected. The targeted sequencing information of 32 myeloid tumor-related genes was successfully collected from 57 individuals using the peripheral blood samples. NGS-based TCR rearrangement data were collected from 73 patients. The last date of follow-up was February, 2024. This study was conducted in compliance with good clinical practice, followed the rules of the Helsinki Declaration, and approved by the Ethics Committee of the First Affiliated Hospital of Nanjing Medical University/Jiangsu Provincial People’s Hospital (ChiCTR2100043485).

Most of the patients were treated with CsA at a starting dose of 5 mg/kg/d, and the level of concentration in serum was adjusted to 150–200 ng/ml according to the adverse effects. Complete remission (CR), partial remission (PR), and non-response (NR) were defined as the achievement of normal Hb levels without transfusion, the presence of anemia without transfusion dependency, and persistent dependence on transfusions, respectively. Overall response rate (ORR) was defined as the percentage of patients with CR or PR.

## Targeted sequencing

Genomic DNA (gDNA) was used to generate the sequencing library by MultipSeq Custom Panel of 32 genes (*ASXL1*, *BCOR*, *BCORL1*, *CALR*, *CBL*, *CEBPA*, *CSF3R*, *DNMT3A*, *ETV6*, *EZH2*, *FLT3*, *GATA2*, *IDH1*, *IDH2*, *JAK2*, *KIT*, *KMT2A*, *KRAS*, *MPL*, *NPM1*, *NRAS*, *PHF6*, *RUNX1*,* SETBP1*, *SF3B1*, *SH2B3*, *SRSF2*, *TET2*, *TP53*, *U2AF1*, *WT1*, *ZRSR2*) (Rightongene Biotechnology Co. Ltd., Shanghai, China) based on the manufacturer’s protocols. The library construction process was as follows: gDNA was amplificated by PCR, and the amplified products were combined and purified by Agencourt AMPure XP magnetic beads (Beckman, California, United States); next, purified products were amplificated by PCR using adapter primers and purified by magnetic beads again; finally, the library concentration was recorded by Qubit dsDNA HS Assay Kit (Thermo, Massachusetts, United States), and the length and purity of the library fragment were measured by Qsep100 automated nucleic acid protein analysis system. The sequencing was performed on the Novaseq (Illumina, United States) sequencing platform. Single nucleotide polymorphisms (SNPs) as well as insertion and deletion (Indels) were screened by Shanghai Rightongene Biotechnology Co., Ltd. (Shanghai, China) based on the following criteria: (1) SNPs or Indels with a mutation allele frequency (MAF) < 0.001 in databases of 1000 genomes project, 1000 genome East Asian, ExAC all or ExAC East Asian and genomAD were retained; (2) SNPs or Indels with a variant allele frequency (VAF) ≥ 1% was retained; (3) the single-nucleotide polymorphism database (dbSNP) (v147) sites existed the Catalog of Somatic Mutations in Cancer (COSMIC) database were retained; (4) missense mutation type require meet the conditions of SIFT score ≤ 0.05 or PolyPhen2 HumanVar database score ≥ 0.447 were retained [20]; (5) SNPs or Indels including stopgain, stoploss, frameshift, nonframeshift and splicing sites were retained.

## NGS sequencing of TCR gene rearrangement

gDNA was extracted from blood samples using QIAamp DNA Tissue Kit (Qiagen, Germantown, MD, USA). NGS-based clonality tests were performed using the TCR Rearrangement Assay Kit (Shanghai Rightongene Biotechnology Co., Ltd., Shanghai, China) according to the manufacturer’s instructions. Briefly, multiplex amplification by PCR was performed using gDNA as a template. After purification by the magnetic bead and quantification by the Qubit fluorometer (Thermo Fisher Scientific, Waltham, MA, USA), libraries were sequenced on an Illumina MiSeq platform (Illumina, San Diego, CA, USA). The amount of sequencing data was 2G. Then, the alignment of sequences was performed by IGBLASTN software. Consensus sequences of the same VDJ classification were counted as one clone, and the corresponding ratio was calculated based on the proportion in the total number of sequences. The frequency of each clonotype in a sample was calculated by dividing the number of sequences reads for each clonotype by the total number of passed sequencing reads in the sample. The clonotypes with a frequency higher than 5% of all rearranged V(D)J sequences were identified as a dominant clone.

### Statistical analysis

The statistical analysis was performed using OriginPro 9.8 software (OriginLab Corporation, Massachusetts, United States). The differences in clinical characteristics and gene mutation frequencies among patients were evaluated using Chi-square test or Fisher’s exact test, as appropriate. The student’s t‑test was performed for comparisons between two groups. Wilcoxon test was applied to continuous variables with nonnormal distribution and adjusted *P* values for multiple tests were calculated by Benjamini-Hochberg procedure. Clonal hematopoiesis risk score (CHRS) was calculated by the CHRS calculator (www.chrsapp.com). Statistical significance was defined as *P* < 0.05.

## Results

### Clinical features of primary PRCA and LGLL-associated PRCA patients

A total of 168 acquired PRCA patients were included in this study, and the clinical information characteristics were summarized in Table 1. Among this cohort, 83 cases were males (49.4%) and 85 were females (50.60%), 89 patients (52.97%) were aged over 60 years, 64 patients were diagnosed with primary PRCA, and 104 patients were diagnosed with LGLL-associated PRCA (LGLL-PRCA). All PRCA patients had dysregulated Hb. No significant differences were observed between the primary PRCA and LGLL-PRCA patients in terms of gender, age, Hb, Ret%, RDW-CV, PLT, MCV, Ferritin, LDH, β2-MG, and Lym%. *STAT3*/*5b* mutations were more likely to occur in primary PRCA patients (*P* = 0.0002). In contrast, compared to primary PRCA patients, LGLL-PRCA patients were more likely to have abnormal ANC (*P* < 0.001). Furthermore, it was found that primary PRCA patients had a higher treatment response rate to CsA than IGLL-associated PRCA (*P* = 0.0079), with a CR rate of 58.5% vs. 33.0% and a PR rate of 18.9% vs. 23.4%.

**Table 1:** Clinical information summary

Characteristic	Total PRCA (*n* = 168)	primary PRCA (*n* = 64)	LGLL-associated PRCA (*n* = 104)	*P* value
Sex [*n*, (%)]				0.7510
Male	83 (49.4)	33 (51.6)	50 (48.1)	
Female	85 (50.6)	31 (48.4)	54 (51.9)	
Age [*n*, (%)]				> 0.9999
< 60	79 (47.0)	30 (46.9)	49 (47.1)	
≥ 60	89 (53.0)	34 (53.1)	55 (52.9)	
Hb [*n*, (%)]				> 0.9999
normal	0 (0.0)	0 (0.0)	0 (0.0)	
dysregulation	168 (100.0)	64 (100.0)	104 (100.0)	
Ret% [*n*, (%)]				0.5270
normal	29 (18.1)	9 (14.8)	20 (20.2)	
dysregulation	131 (81.9)	52 (85.2)	79 (79.8)	
(missing, *n*)	8	3	5	
RDW-CV [*n*, (%)]				0.0746
normal	83 (50.9)	37 (60.7)	46 (45.1)	
dysregulation	80 (49.1)	24 (39.3)	56 (54.9)	
(missing, *n*)	5	3	2	
ANC [*n*, (%)]				< 0.001
normal	107 (64.1)	63 (100.0)	44 (42.3)	
dysregulation	60 (35.9)	0 (0.0)	60 (57.7)	
(missing, *n*)	1	1	0	
PLT [*n*, (%)]				0.2200
normal	120 (71.4)	42 (65.6)	78 (75.0)	
dysregulation	48 (28.6)	22 (34.4)	26 (25.0)	
MCV [*n*, (%)]				0.7370
normal	109 (66.5)	43 (68.3)	66 (65.3)	
dysregulation	55 (33.5)	20 (31.7)	35 (34.7)	
(missing, *n*)	4	1	3	
Ferritin [*n*, (%)]				0.1300
normal	29 (21.0)	7 (13.5)	22 (25.6)	
dysregulation	109 (79.0)	45 (86.5)	64 (74.4)	
(missing, *n*)	30	12	18	
LDH [n, (%)]				0.5910
normal	101 (67.3)	38 (70.4)	63 (65.6)	
dysregulation	49 (32.7)	16 (29.6)	33 (34.4)	
(missing, *n*)	18	10	8	
β2-MG [*n*, (%)]				0.5040
normal	60 (60.0)	20 (66.7)	40 (57.1)	
dysregulation	40 (40.0)	10 (33.3)	30 (42.9)	
(missing, *n*)	68	34	34	
MF [*n*, (%)]				0.0172
No	48 (70.6)	25 (86.2)	23 (59.0)	
Yes	20 (29.4)	4 (13.8)	16 (41.0)	
(missing, *n*)	100	35	65	
*STAT3/5b* mutation [*n*, (%)]				0.0002
Yes	100 (79.4)	33 (100.0)	67 (72.0)	
No	26 (20.6)	0 (0.0)	26 (28.0)	
(missing, *n*)	42	31	11	
Lym% [*n*, (%)]				0.2490
normal	75 (46.9)	31 (53.4)	44 (43.1)	
dysregulation	85 (53.1)	27 (46.6)	58 (56.9)	
(missing, *n*)	8	6	2	
CD3 + CD8+/Lym% [*n*, (%)]				0.0004
normal	38 (25.2)	23 (42.6)	15 (15.5)	
dysregulation	113 (74.8)	31 (57.4)	82 (84.5)	
(missing, *n*)	17	10	7	
CD3 + CD4+/Lym% [*n*, (%)]				0.0197
normal	51 (33.8)	25 (46.3)	26 (26.8)	
dysregulation	100 (66.2)	29 (53.7)	71 (73.2)	
(missing, *n*)	17	10	7	
CsA treatment				
Complete remission	62 (42.2)	31 (58.5)	31 (33.0)	0.0079
Partial remission	32 (21.8)	10 (18.9)	22 (23.4)	
Non-response	53 (36.0)	12 (22.6)	41 (43.6)	

Hb, hemoglobin; Ret%, reticulocyte percentage; RDW-CV, red cell distribution width-coefficient of variation; ANC, absolute neutrophil count; PLT, platelet count; MCV, mean corpuscular volume; LDH, lactic dehydrogenase; β2-MG, beta 2-macroglobulin; MF, myelofibrosis; Lym%, lymphocyte percentage; CsA, cyclosporine

## Mutation landscape of primary PRCA and LGLL-PRCA patients

**Fig. 1:**
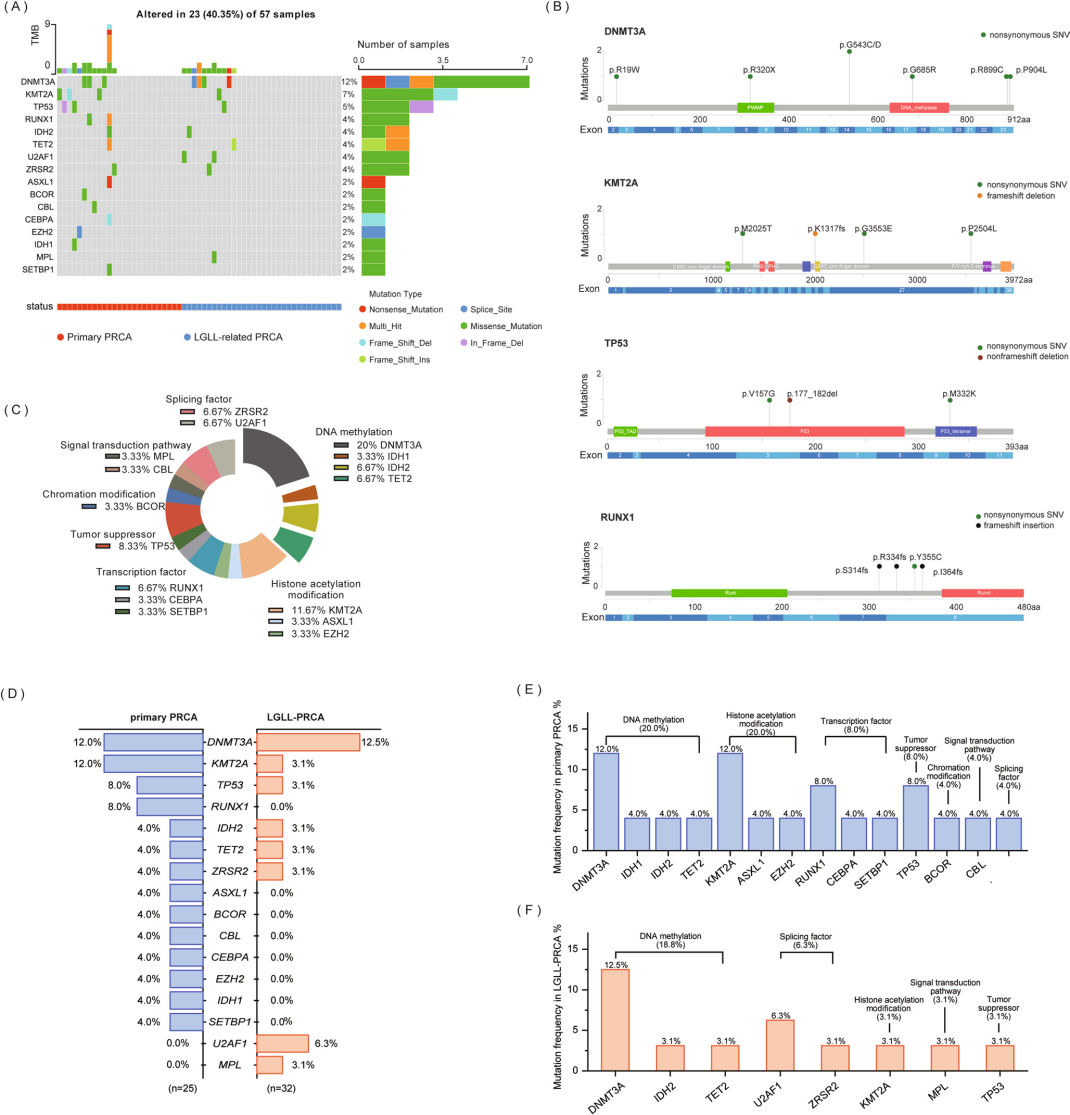
Overview of mutations in primary PRCA and LGLL-associated PRCA patients. (**A**) Landscape of gene mutation in PRCA; (**B**) Lollipop plot for DNMT3A, KMT2A, TP53 and RUNX1 mutations in PRCA cohort; (**C**) The distribution of gene mutations in different pathways; (**D**) The frequency of mutated genes in primary PRCA and LGLL-associated PRCA patients; (**E**) Gene mutation frequency and enrichment pathway in primary PRCA patients; (**F**) Gene mutation frequency and enrichment pathway in LGLL-associated PRCA patients

57 patients, including 25 cases with primary PRCA and 32 LGLL-PRCA cases had available targeted sequencing data, with 23 individuals (40.35%) carried at least one gene mutation. The most frequently mutated genes were *DNMT3A* and *KMT2A*. *TP53*, *IDH2*, *RUNX1*, *TET2*, *U2AF1* and *ZRSR2* were identified as the second most frequently mutated genes (Fig. 1A). Missense mutations were the predominant variant type, detected in 16 patients (28.1%). Among the genes that most frequently undergo mutations, six types of amino acid (aa) variations were identified in *DNMT3A* with two mutations at aa 543 (p.G543C/D); four mutation sites of *KMT2A* were detected with a frameshift deletion at aa 1317 (p.K1317fs); three mutation sites of *TP53* were detected in PRCA patients with a nonframeshift deletion (p.177_182del); and four mutation sites of *RUNX1* were detected with three frameshift insertion (Fig. 1B). *DNMT3A*, *IDH1*, *IDH2*, and *TET2* belongs to the DNA methylation signaling, with the total mutation frequency of this signaling was 36.67%. *KMT2A*, *ASXL1*, and *EZH2* were associated with histone acetylation modification, and the total mutation frequency relating to this signaling was 18.33%. *BCOR* was associated with chromatin modification (3.33%); *MPL* and *CBL* were associated with signal transduction pathway (6.66%); *TP53* was a tumor suppressor (8.33%); *ZRSR2* and *U2AF1* were splicing factors (13.34%); and *RUNX1*, *CEBPA* and *SETBP1* were transcription factors (13.33%) (Fig. 1C).

Based on the statistical results, the mutation frequency of *DNMT3A* was the highest in both two cohorts (Fig. 1D). Mutations in *RUNX1*, *ASXL1*, *BCOR*, *CBL*, *CEBPA*, *EZH2*, *IDH1* and *SETBP1* were only found in patients with primary PRCA, and mutations in *U2AF1* and *MPL* were only found in patients with LGLL-PRCA (Fig. 1D). Mutations in *BCORL1*, *CALR*, *CSF3R*, *ETV6*, *FLT3*, *GATA2*, *JAK2*, *KIT*, *KRAS*, *NPM1*, *NRAS*, *PHF6*, *SF3B1*, *SH2B3*, *SRSF2* and *WT1* genes were not detected in neither primary PRCA or LGLL-PRCA groups.

In order to better analyze the potential function affected by these mutations, the overall mutation frequency of the pathway was calculated by integrating gene mutations belonging to the same pathway. Gene mutations in primary PRCA patients were mainly enriched in DNA methylation (20.0%) and histone acetylation modification (20.0%) (Fig. 1E), while gene mutations in LGLL-PRCA patients were mainly enriched in DNA methylation (18.8%) and splicing factors (6.3%) (Fig. 1F). The frequency of epigenetic-related gene mutations (*ASXL1*, *DNMT3A*, *EZH2*, *IDH1*, *IDH2*, *KMT2A* and *TET2*) showed no significant difference between the primary PRCA group and the LGLL-PRCA group (*P* = 0.37).

**Fig. 2:**
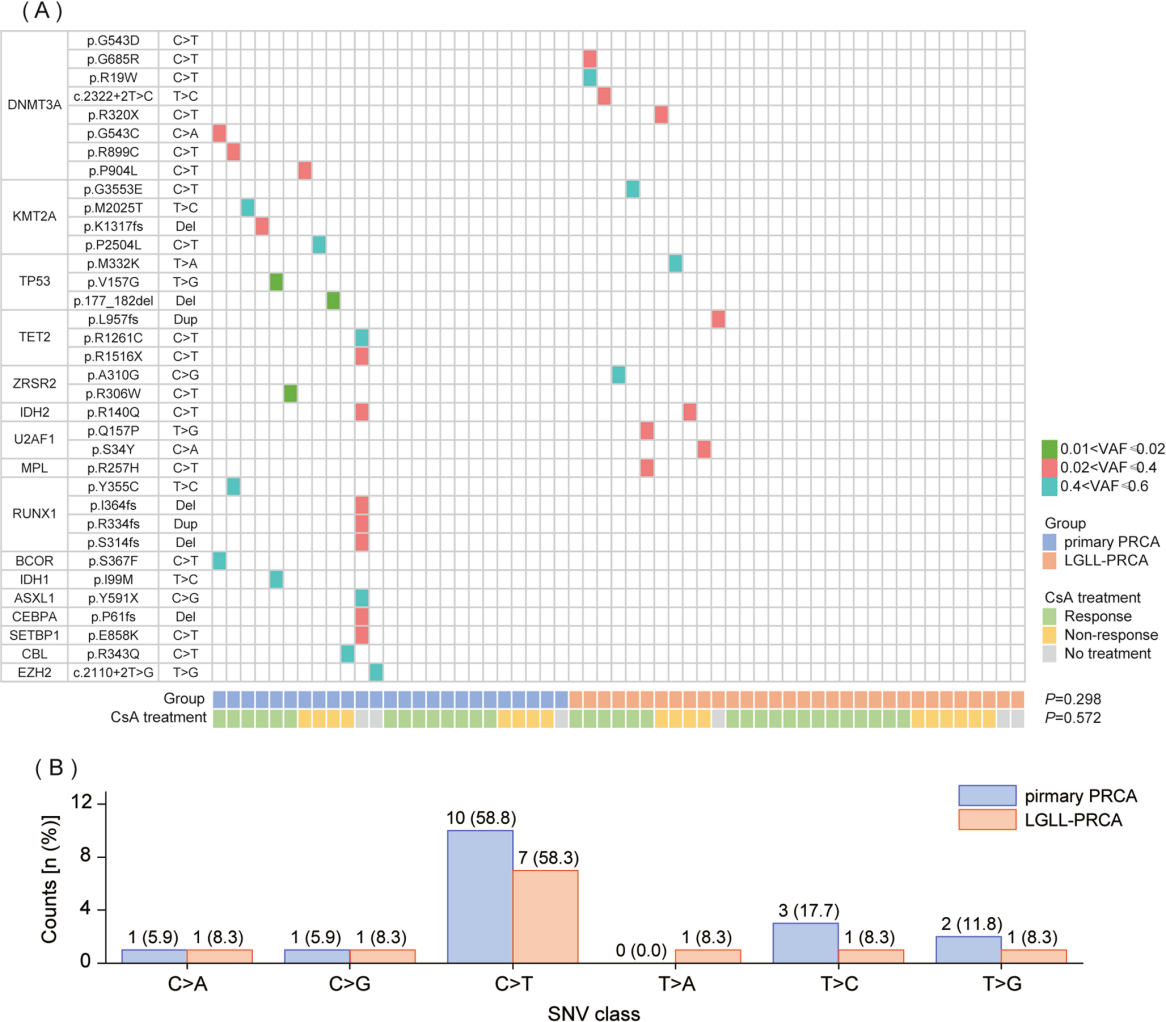
Overview of mutated sites in primary PRCA and LGLL-associated PRCA patients. (**A**) The mutation information of primary PRCA and LGLL-associated PRCA patients; (**B**) The single nucleotide variants (SNV) class of primary PRCA and LGLL-associated PRCA patients

Notably, we found that although the primary PRCA and LGLL-PRCA groups had the same mutated genes, gene mutation sites were completely discordant in these two groups except for *IDH2* (Fig. 2A). The VAF of mutations in patients with acquired PRCA was mainly in the range of 0.02 to 0.4 (Fig. 2A).

The proportion of T to adenine (A) base pair substitutions was 8.3% in LGLL-PRCA patients, but this alteration was not detected in the primary PRCA patients (Fig. 2B). It was also found that base pair deletion-associated frame shift mutations occurred only in primary PRCA patients (80%), whereas frame shift mutations in LGLL-PRCA patients were mostly associated with base pair insertions (100%) (Fig. 2A).

## TCR rearrangement profile of LGLL-PRCA patients

**Fig. 3:**
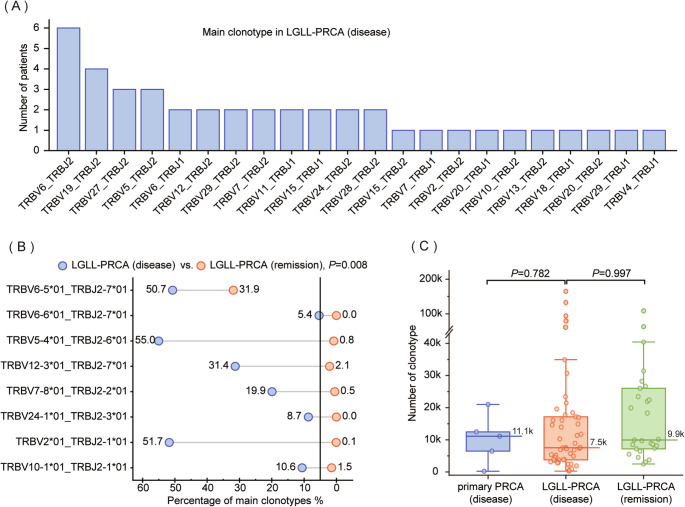
TCR rearrangement assessment of LGLL-associated PRCA. (**A**) Main clonotypes (> 5% in percentage) in LGLL-associated PRCA patients at disease stage; (**B**) Changes in percentage of the main clonotypes for the same LGLL-associated PRCA patient from disease stage to remission stage; (**C**) The number of clonotypes in different periods of patients

8 primary PRCA patients and 65 LGLL-PRCA patients were subjected to NGS-based TCR rearrangement testing. Dominant clonotype (with > 5% clone) was detected in 1 patient with primary PRCA and 58 patients with LGLL-PRCA. Interestingly, some clonotypes were frequently occurred, including TRBV6_TRBJ2, TRBV19_TRBJ2, TRBV5_TRBJ2 and TRBV27_TRBJ2 in LGLL-PRCA cohort (Fig. 3A). In the ongoing follow-up of 8 LGLL-PRCA patients, it was found that the percentage of the same dominant clonotype was clearly decreased in remission period (Disease vs. Remission, *P* = 0.0081, Fig. 3B). The median number of clonotypes was lower in the LGLL-PRCA disease status (7541 clonotypes), than that in the primary PRCA disease status (11097 clonotypes) and LGLL-PRCA remission status (9954 clonotypes) (Fig. 3C). These results illustrated the potential significance of TCR rearrangements for disease course.

### Clinical correlation with mutations and TCR rearrangement in primary PRCA and LGLL-PRCA patients

**Fig. 4:**
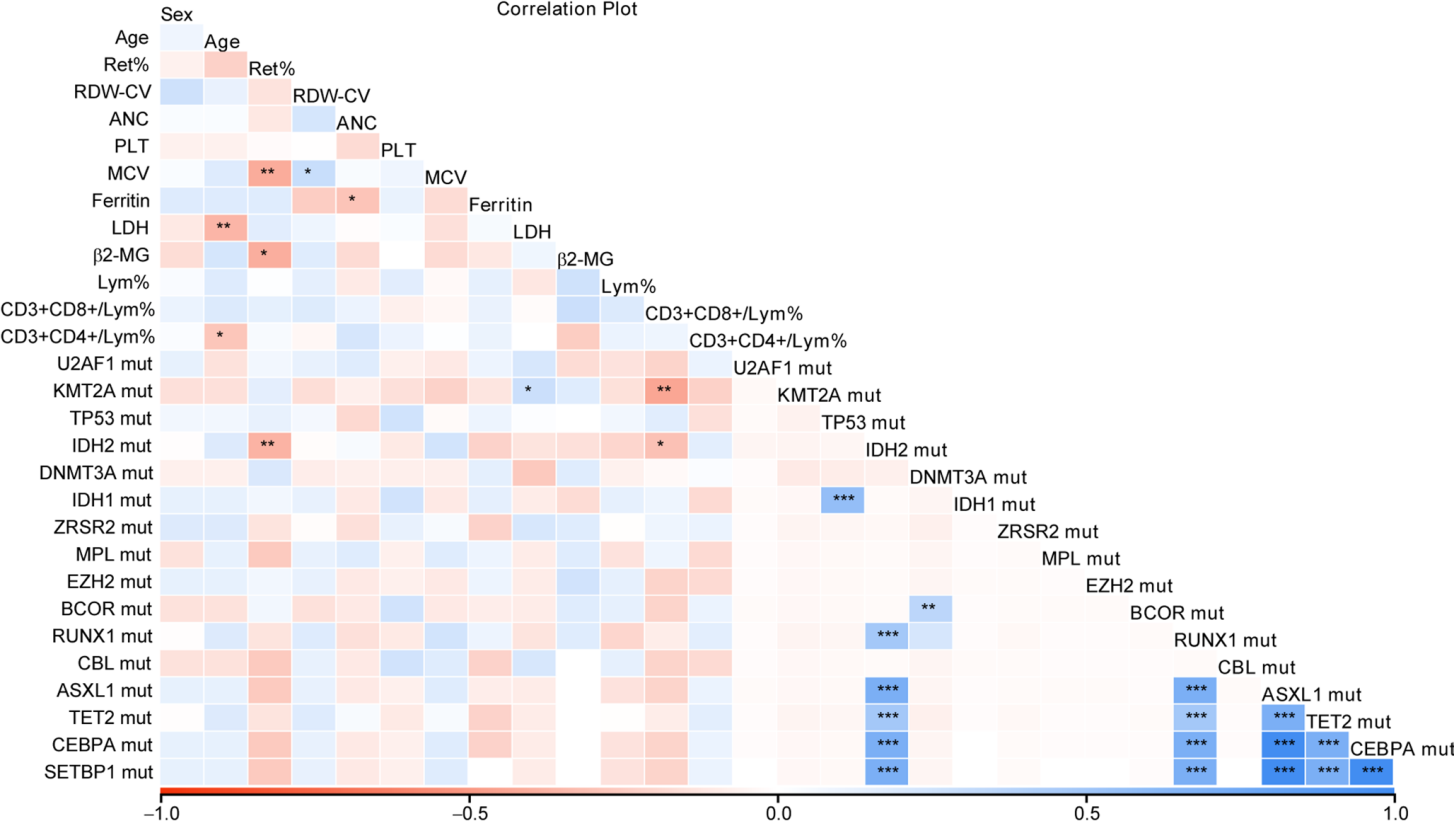
Correlation of clinical information with mutation in PRCA patients. Correlation plot of clinical information with mutation in PRCA patient

In addition, we analyzed the correlation of clinical information with mutations and TCR rearrangements in different groups of PRCA patients. The results showed that gene mutations were enriched in patients who were older than 40 years, while no significant difference was found in the proportion of mutation carrier and median VAF of clonal hematopoietic-related mutations (Table 2). Correlation analysis showed that Ret% was closely associated with *IDH2* mutation, LDH was significantly associated with *KMT2A* mutation, and CD3 + CD8+/Lym% was strongly associated with *KMT2A* and *IDH2* mutation (Fig. 4). Further Fisher’s exact test showed tha*t IDH2* mutation was more common in patients with normal Ret% level (2/12 vs. 0/44, *P* = 0.0429), and *KMT2A* mutation was enriched in patients with normal CD3 + CD8+/Lym% (4/16 vs. 0/38, *P* = 0.0058).

**Table 2:** Gene mutations in primary PRCA patients and LGLL-associated PRCA patients of different ages

Groups	primary PRCA patients				LGLL-associated PRCA patients			
	Age < 40 (*n* = 2)	40 ≤ Age < 60 (*n* = 11)	Age ≥ 60 (*n* = 12)	*P* value	Age < 40 (*n* = 1)	40 ≤ Age < 60 (*n* = 13)	Age ≥ 60 (*n* = 18)	*P* value
Mutation [n, (%)]	0 (0.0)	6 (54.5)	6 (50.0)	0.36	0 (0.0)	4 (30.8)	7 (38.9)	0.68
VAF [Median (ranges)]	/	0.481 (0.026–0.504)	0.082 (0.011–0.569)	0.33	/	0.205 (0.066–0.474)	0.257 (0.056–0.498)	0.51
*DNMT3A* mutation [n, (%)]	0 (0.0)	2 (18.2)	1 (8.3)	0.48	0 (0.0)	2 (15.4)	2 (11.1)	0.73
*TET2* mutation [n, (%)]	0 (0.0)	0 (0.0)	1 (8.3)	0.33	0 (0.0)	0 (0.0)	1 (5.6)	0.39
*ASXL1* mutation [n, (%)]	0 (0.0)	0 (0.0)	1 (8.3)	0.33	0 (0.0)	0 (0.0)	0 (0.0)	/

Also, we compared the differences of clinicopathological information between patients carrying mutation or not. In primary PRCA patients, a significant different was observed between normal CD3 + CD8+/Lym% and patients with gene mutation (*P* = 0.0123, Table 3). A negative correlation between *KMT2A* mutation and CD3 + CD8+/Lym% was also found in the primary PRCA cohort (Supplementary Fig. 1A), but further Fisher’s exact test result showed no significant (Supplementary Fig. 1A). No significant difference was observed in clinical information among LGLL-PRCA patients with or without mutation (Table 4). The correlation analysis revealed significant correlation between PLT and *TP53* mutation, CD3 + CD8+/Lym% and *KMT2A* mutation, as well as a correlation between CD3 + CD8+/Lym% and *IDH2* mutation, while the subsequent Fisher’s exact test results were not significant (Supplementary Fig. 1B). In addition, the result showed no correlation between the gene mutation and treatment response to CsA (primary PRCA with gene mutation vs. without gene mutation, *P* > 0.05 in Table 3; LGLL-PRCA with gene mutation vs. without gene mutation, *P* > 0.05 in Table 4).

**Table 3:** Clinical information in primary PRCA patients with or without gene mutations

Characteristic	primary PRCA with gene mutation (*n* = 12)	primary PRCA without gene mutation (*n* = 13)	*P* value (mutation vs. no mutation)
Sex (*n*)			0.2380
male	5 (41.7)	9 (69.2)	
female	7 (58.3)	4 (30.8)	
Age [*n*, (%)]			> 0.9999
< 60	6 (50)	7 (53.8)	
≥ 60	6 (50)	6 (46.2)	
Hb [*n*, (%)]			> 0.9999
normal	0 (0.0)	0 (0.0)	
dysregulation	12 (100.0)	13 (100.0)	
Ret% [*n*, (%)]			> 0.9999
normal	2 (16.7)	2 (16.7)	
dysregulation	10 (83.3)	10 (83.3)	
(missing, *n*)	0	1	
RDW-CV [*n*, (%)]			0.6950
normal	6 (50.0)	8 (61.5)	
dysregulation	6 (50.0)	5 (38.5)	
ANC [*n*, (%)]			> 0.9999
normal	12 (100.0)	13 (100.0)	
dysregulation	0 (0.0)	0 (0.0)	
PLT [*n*, (%)]			> 0.9999
normal	8 (66.7)	9 (69.2)	
dysregulation	4 (33.3)	4 (30.8)	
MCV [*n*, (%)]			0.4280
normal	8 (66.7)	6 (46.2)	
dysregulation	4 (33.3)	7 (53.8)	
Ferritin [*n*, (%)]			0.5870
normal	3 (27.3)	1 (9.1)	
dysregulation	8 (72.7)	10 (90.9)	
(missing, *n*)	1	2	
LDH [n, (%)]			> 0.9999
normal	8 (72.7)	8 (66.7)	
dysregulation	3 (27.3)	4 (33.3)	
(missing, *n*)	1	1	
β2-MG [*n*, (%)]			0.6080
normal	3 (60.0)	4 (40.0)	
dysregulation	2 (40.0)	6 (60.0)	
(missing, *n*)	7	3	
MF [n, (%)]			> 0.9999
normal	7 (100.0)	7 (87.5)	
dysregulation	0 (0.0)	1 (12.5)	
(missing, *n*)	5	5	
Lym% [*n*, (%)]			0.4140
normal	7 (58.3)	4 (33.3)	
dysregulation	5 (41.7)	8 (66.7)	
(missing, *n*)	0	1	
CD3 + CD8+/Lym% [*n*, (%)]			0.0123
normal	8 (72.7)	2 (16.7)	
dysregulation	3 (27.3)	10 (83.3)	
(missing, *n*)	1	1	
CD3 + CD4+/Lym% [*n*, (%)]			0.4140
normal	7 (63.6)	5 (41.7)	
dysregulation	4 (36.4)	7 (58.3)	
(missing, *n*)	1	1	
CsA treatment			0.6668
Complete remission	6 (60.0)	7 (58.3)	
Partial remission	0 (0.0)	1 (8.3)	
Non-response	4 (40.0)	4 (33.3)	
(missing, *n*)	2	1	

Hb, hemoglobin; Ret%, reticulocyte percentage; RDW-CV, red cell distribution width-coefficient of variation; ANC, absolute neutrophil count; PLT, platelet count; MCV, mean corpuscular volume; LDH, lactic dehydrogenase; β2-MG, beta 2-macroglobulin; MF, myelofibrosis; Lym%, lymphocyte percentage; CsA, cyclosporine

**Table 4:** Clinical information in LGLL-associated PRCA patients with or without gene mutations

Characteristic	LGLL-associated PRCA with gene mutation (*n* = 11)	LGLL-associated PRCA without gene mutation (*n* = 21)	*P* value (mutation vs. no mutation)
Sex [*n*, (%)]			> 0.9999
Male	6 (54.5)	10 (47.6)	
Female	5 (45.5)	11 (52.4)	
Age [*n*, (%)]			0.7120
< 60	4 (36.4)	10 (47.6)	
≥ 60	7 (63.6)	11 (52.4)	
Hb [*n*, (%)]			> 0.9999
normal	0 (0.0)	0 (0.0)	
dysregulation	11 (100.0)	21 (100.0)	
Ret% [*n*, (%)]			> 0.9999
normal	3 (27.3)	5 (23.8)	
dysregulation	8 (72.7)	16 (76.2)	
RDW-CV [*n*, (%)]			> 0.9999
normal	4 (36.3)	8 (38.1)	
dysregulation	7 (63.6)	13 (61.9)	
ANC [*n*, (%)]			0.7026
normal	4 (36.4)	6 (28.6)	
dysregulation	7 (63.6)	15 (71.4)	
PLT [*n*, (%)]			0.6370
normal	10 (91.0)	17 (81.0)	
dysregulation	1 (9.0)	4 (19.0)	
MCV [*n*, (%)]			> 0.9999
normal	7 (63.6)	14 (66.7)	
dysregulation	4 (36.4)	7 (33.3)	
Ferritin [*n*, (%)]			0.2310
normal	2 (22.2)	9 (50.0)	
dysregulation	7 (77.8)	9 (50.0)	
(missing, *n*)	2	3	
LDH [n, (%)]			0.6750
normal	6 (60.0)	14 (73.7)	
dysregulation	4 (40.0)	5 (26.3)	
(missing, *n*)	1	2	
β2-MG [n, (%)]			0.6590
normal	3 (42.9)	8 (57.1)	
dysregulation	4 (57.1)	6 (42.9)	
(missing, *n*)	4	7	
MF [*n*, (%)]			0.2870
normal	3 (42.9)	10 (83.3)	
dysregulation	4 (57.1)	2 (16.7)	
(missing, *n*)	4	9	
Lym% [*n*, (%)]			> 0.9999
normal	6 (54.5)	10 (47.6)	
dysregulation	5 (45.5)	11 (52.4)	
CD3 + CD8+/Lym% [*n*, (%)]			0.3580
normal	3 (30.0)	3 (14.3)	
dysregulation	7 (70.0)	18 (85.7)	
(missing, n)	1	0	
CD3 + CD4+/Lym% [*n*, (%)]			> 0.9999
normal	3 (30.0)	5 (23.8)	
dysregulation	7 (70.0)	16 (76.2)	
(missing, *n*)	1	0	
CsA treatment			0.6605
Complete remission	3 (30.0)	9 (47.4)	
Partial remission	3 (30.0)	4 (21.0)	
Non-response	4 (40.0)	6 (31.6)	
(missing, *n*)	1	2	

Hb, hemoglobin; Ret%, reticulocyte percentage; RDW-CV, red cell distribution width-coefficient of variation; ANC, absolute neutrophil count; PLT, platelet count; MCV, mean corpuscular volume; LDH, lactic dehydrogenase; β2-MG, beta 2-macroglobulin; MF, myelofibrosis; Lym%, lymphocyte percentage; CsA, cyclosporine

### Factors influencing csa response in PRCA patients

Recently, researchers have found that CHRS is a potent tool to predict the risk of myeloid malignancy [21], herein, we evaluated whether CHRS associated with the CsA response. Primary PRCA patients were grouped into low-risk group (*n* = 17), intermediate-risk group (*n* = 4), and high-risk group (*n* = 4). LGLL-PRCA patients were grouped into low-risk group (*n* = 25), intermediate-risk group (*n* = 1), and high-risk group (*n* = 5). There was no significant difference in the composition of risk categorization (low vs. intermediate + high) between primary PRCA patients and LGLL-PRCA patients (primary PRCA vs. LGLL-PRCA, *P* = 0.36). Meanwhile, no significant relationship between risk categorization and response to CsA treatment was found (Table 5).

**Table 5:** Relationship between clonal hematopoiesis risk score (CHRS) and csa response

Risk categories	primary PRCA patients				LGLL-associated PRCA patients			
	Low risk (*n* = 17)	Intermediate risk (*n* = 4)	High risk (*n* = 4)	*P* value	Low risk (*n* = 25)	Intermediate risk (*n* = 1)	High risk (*n* = 5)	*P* value
Complete remission	10 (62.5)	1 (33.3)	2 (66.7)	0.799^a^ 0.451^b^	12 (52.2)	0 (0)	0 (0)	0.096^a^ 0.369^b^
Partial remission	1 (6.3)	0 (0)	0 (0)		4 (17.4)	0 (0)	3 (60.0)	
Non-response	5 (31.3)	2 (66.7)	1 (33.3)		7 (30.4)	1 (100)	2 (40.0)	
(missing, *n*)	1	1	1		2	0	0	

Clonal Hematopoiesis Risk Score (CHRS) Calculator, www.chrsapp.com. ^a^, comparison carried out between 3 classifications, complete remission, partial remission and non-response; ^b^, comparison carried out between 2 classifications, response (complete remission, partial remission) and non-response

**Fig. 5:**
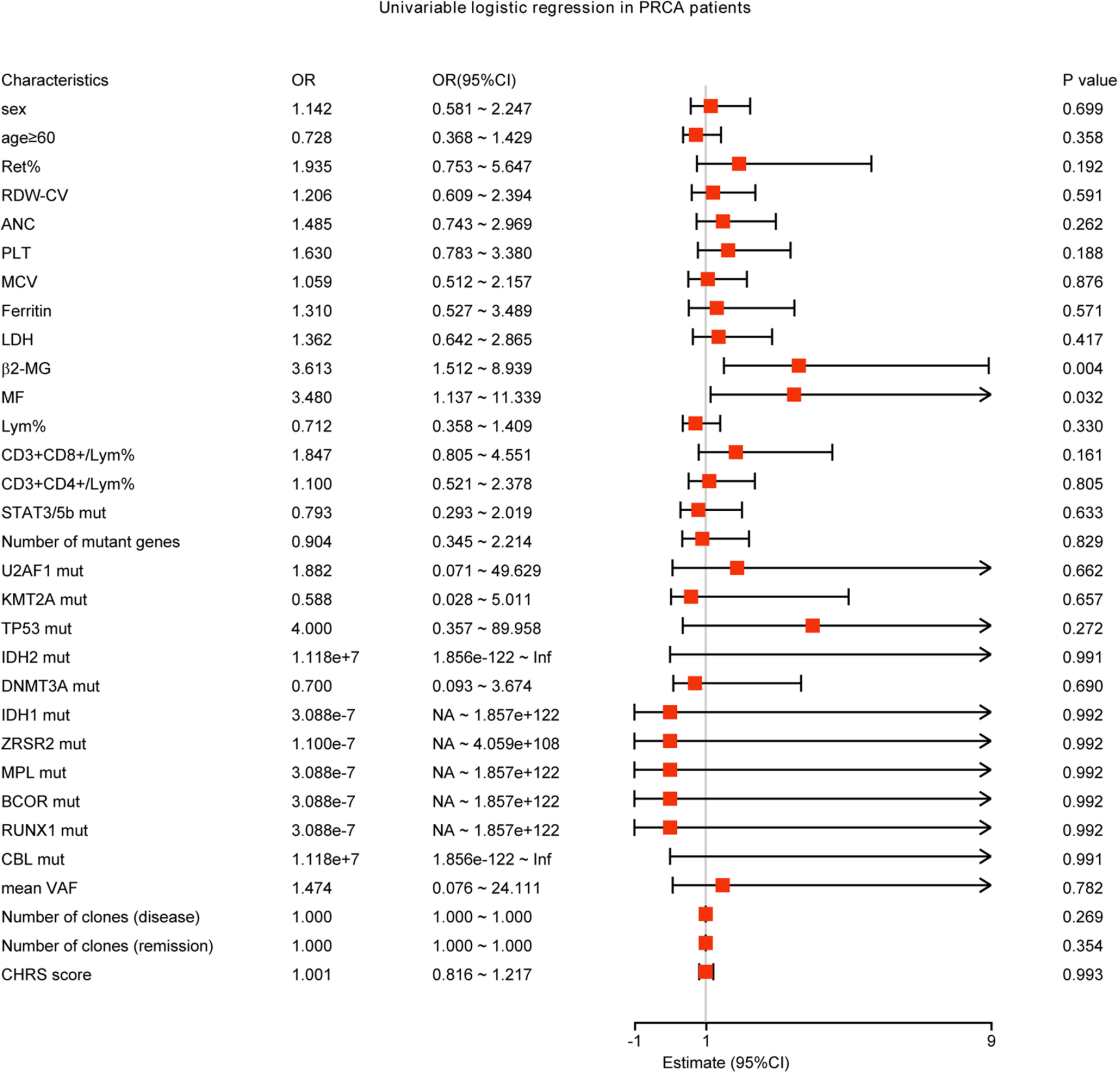
Univariable logistic regression in PRCA patients

The univariable logistic regression results showed that β2-MG dysregulation and MF dysregulation were unfavorable factors for the efficacy of CsA treatment in PRCA patients (*P* = 0.004, *P* = 0.032), and other clinical diagnostic information, mutated genes, number of mutated genes, mean VAF, number of TCR clones in PRCA patients did not significantly affect CsA treatment response (*P* > 0.05, Fig. 5). Separately, PLT dysregulation might be an unfavorable factor affecting CsA efficacy in primary PRCA patients (*P* = 0.05, Supplementary Fig. 2A), and β2-MG dysregulation was an unfavorable factor affecting CsA efficacy in LGLL-PRCA patients (*P* = 0.033, Supplementary Fig. 2B).

## Discussion

PRCA is a rare disease that can be categorized as congenital or acquired based on the pathogenesis [2]. However, the diverse etiologies, as well as low incidence of acquired PRCA make its pathogenesis remain largely unclear [15]. Herein, we portrayed the clinicopathologic, mutation and TCR rearrangement profiles of 64 primary PRCA cases and 104 LGLL-associated PRCA. Among the 168 patients, the male and female ratio is 0.98 (83/85), which was higher than that of Thibile’s study (5/22) [22]. The population heterogeneity may cause this difference as 96.3% patients were infected with HIV in Thibile’s study. Immune status plays a key role in the pathogenesis of both primary and secondary PRCA [13]. Herein, we found that the ANC dysregulation was only occurred in the LGLL-related PRCA cohort. We believe that neutropenia is the result of an LGLL-mediated autoimmune attack, rather than the key players in LGLL pathogenesis, since Western patients with LGLL frequently present with neutropenia or even agranulocytosis, whereas these conditions are seldom observed in Asian patients [23]. Our results may support the role of ANC in distinguish LGLL-PRCA and primary PRCA, but need to be further validation. In addition, we found MF was more common in the LGLL-PRCA compared with the primary PRCA cases, which may also help in LGLL-PRCA diagnosis.

In terms of gene mutation profile, we found that gene mutations detected in primary PRCA patients mainly enriched in DNA methylation, histone acetylation modification, transcription factor, and tumor suppressor pathways; and the mutations in LGLL-associated PRCA patients located on genes related to DNA methylation and splicing factor signals, which was similarly to Long’s study [15]. Based on the detected gene panel, the most frequent 3 mutation genes were *DNMT3A*, *KMT2A* and *TP53*, which were also reported in previous studies with similar mutation frequency [13, 18, 24]. *RUNX1*, *ASXL1*, *BCOR*, *CBL*, *CEBPA*, *EZH2*, *IDH1* and *SETBP1* mutation were only detected in primary PRCA patients, while mutations in *U2AF1*, a splicing factor-associated gene, and *MPL*, a signaling transduction pathway-associated gene, were only detected in LGLL-associated PRCA patients, demonstrating the different pathogenesis of primary PRCA and LGLL-PRCA. Studies have reported that genes such as *U2AF1* and *MPL*, which associated with myeloid malignancies, were relatively rare in PRCA [25–27]. Evidence has suggested that clonal hematopoiesis may exhibit variations among different races [28, 29]. In our study, approximately 40% of PRCA patients exhibited clonal hematopoiesis. In two other East Asian patient cohorts, clonal hematopoiesis was identified in as high as 53% [15] and 62% [30] of the patients. Additionally, *DNMT3A*, *TET2*, and *BCOR*, which occurred at relatively high frequencies in these cohorts, were also recurrent in our cohort. In contrast, a study cohort composed of American patients found no somatic mutations typical of myeloid clonal hematopoiesis [18]. This suggests that the distribution of clonal hematopoiesis in PRCA patients may also be associated with ethnicity. In addition, our results show parallels between acquired PRCA and aplastic anemia. The conversion of C to T was the major source of nucleotide alterations in acquired PRCA [31]. Similar C-to-T conversion mutations accumulate in hematopoietic progenitors in healthy persons [32]. We also found that the VAF of gene mutations was mainly enriched in the range of 0.02 to 0.4, which was consistent with the previous study, suggesting that the incidence of clonal hematopoiesis was higher among patients with acquired PRCA patients [13].

Further, we analyzed the correlation between mutation landscape and clinical features. It has been reported that age-related clonal hematopoiesis was identified in 1 to 3% of patients with nonhematologic cancers and in healthy persons, and was associated with a high risk of the development of subsequent hematologic malignancies [32–35]. In this study, we detected gene mutations only in patients older than 40 years, and the percentage of mutations occurring in patients older than 60 years was higher than in patients between 40 and 59 years of age. These observations are in consistent with the natural pattern of clonal hematopoiesis increasing with age [36], and might be independent of PRCA pathogenesis. For primary PRCA, patients carrying gene mutations had significantly lower Hb than those not carrying mutations. Meanwhile, the *ZRSR2* mutation had a potential effect on the increase of ANC. Such results seem to be consistent with the previous reports [37, 38]. In this PRCA cohort, we found an interesting phenomenon that *KMT2A* mutation was only detected in patients with normal Ret%, while *IDH1* mutation only occurred in patients with normal CD3 + CD8+/Lym%. These findings indicate a link between gene mutation and clinical features, which may contribute to exploring the pathogenesis of PRCA.

The treatment of PRCA is challenging. Although our single-center study, limited by the small sample size, did not identify a statistically significant difference in treatment response between CsA and CS [6, 9], a systematic review and meta-analysis revealed a significant advantage of CsA over CS as a first-line treatment in terms of ORR (74% vs. 47%) [39]. Zhang et al. [40] compared the efficacy and safety of CsA and CsA + CS regimen in 96 patients. No significant difference was found between the two groups in the optimal ORR, optimal CR rate (CRR), time to response or time to CR. CsA + CS and CsA groups had similar adverse event (AE) rates, but CsA + CS group had higher CS-related infection rate. Herein, 147 patients received CsA treatment, achieving a CRR of 42.2% and an ORR of 64%. The response rate of LGLL-associated PRCA to CsA treatment was lower than primary PRCA (*P* = 0.001) [6]. A similar result was found in our cohort with an ORR of 77.4% in primary PRCA and 56.4% in the LGLL-PRCA. Also, we tried to reveal the influencing factor for the response to CsA in this cohort. The logistic regression results showed that β2-MG dysregulation and MF dysregulation were unfavorable factors for the response to CsA in PRCA patients. Consistently, age, gender, Hb level at diagnosis, and gene mutation or not showed no influence on the response to immunosuppressive therapy, as reported by Long et al. [40]. The CHRS calculator was also used to categorize the acquired PRCA patients, but no significant relationship between risk categorization and response to CsA treatment was found. In fact, these results maybe need to be validated with a larger sample size and may lead to exciting findings. In addition, the results of the clonal diversity analysis showed that the number of TCR clonotypes was obviously lower in LGLL-associated PRCA patients during disease stage compared with primary PRCA patients of disease stage and LGLL-associated PRCA patients of remission stage, which might be related to the clonal diversity of LGLL [41].

Some limitations should be stated of the current study. One is the incomplete molecular profiling data​​. Comprehensive molecular profiling was not available across the cohort. This data gap may limit the holistic interpretation of tumor heterogeneity and immune microenvironment interactions. Second is the absence of longitudinal paired mutation data of LGLL​​. For patients with LGLL, ​​longitudinally paired tumor samples​​ during active disease and remission phases were unavailable. This precluded temporal correlation between mutational evolution (e.g., clonal drift) and dynamic TCR repertoire changes. We intend to play multi-center collaborations with multi-omics using serial sampling (e.g., paired blood/bone marrow at diagnosis, remission, and relapse) to further clarify the pathogenesis of PRCA.

In summary, the current study systematically described the clinical features, mutation landscape and TCR rearrangement profile in a relative larger PRCA cohort, as well as tried to reveal the association factors for the response to CsA. Meanwhile, the study found that gene mutations were detected in 39.7% of acquired PRCA who were older than 40 years, with *DNMT3A*, *KMT2A* and *TP53* being the top three mutated genes. For LGLL-associated PRCA patients, TRBV6_TRBJ2 was the most frequent dominant clonotype and the proportion of each dominant clone decreased following the remission of anemia. The response rate of LGLL-associated PRCA to CsA treatment was lower than primary PRCA. β2-MG dysregulation and MF dysregulation were unfavorable factors for the response to CsA in PRCA patients, while other clinical diagnostic information, mutated genes, number of mutated genes, mean VAF, number of TCR clones in PRCA patients did not significantly affect the response to CsA.

## Supplementary Information

Below is the link to the electronic supplementary material.

Supplementary figure 1 Correlation of clinical information with mutation and clonal diversity in primary PRCA patients and LGLL-PRCA patients. (A) Correlation plot of clinical information with mutation in primary PRCA patient; (B) Correlation plot of clinical information with mutation in LGLL-PRCA patient. (PNG 320 KB)

High Resolution Image (TIF 1.63 MB)

Supplementary figure 2 Univariable logistic regression in primary PRCA and LGLL-PRCA patients. (A) Univariable logistic regression in primary PRCA patients; (B) Univariable logistic regression in LGLL-PRCA patients. (PNG 205 KB)

High Resolution Image (TIF 1.76 MB)

## Data Availability

The data that support the findings of this study are available from the corresponding author upon reasonable request.

## References

[1] Means RT Jr (2016) Pure red cell aplasia. Blood 128(21):2504–2509. 10.1182/blood-2016-05-717140 27881371 10.1182/blood-2016-05-717140

[2] Means RT Jr. (2023) Pure red cell aplasia: the second hundred years. Am J Med Sci 366(3):160–166. 10.1016/j.amjms.2023.06.009 10.1016/j.amjms.2023.06.009 37327996

[3] Means RT, Jr. (2016) Pure red cell aplasia. Hematol Am Soc Hematol Educ Program 2016 1:51–56. 10.1182/asheducation-2016.1.51 10.1182/asheducation-2016.1.51 PMC6142432 27913462

[4] Sawada K, Fujishima N, Hirokawa M (2008) Acquired pure red cell aplasia: updated review of treatment. Br J Haematol 142(4):505–514. 10.1111/j.1365-2141.2008.07216.x 18510682 10.1111/j.1365-2141.2008.07216.x PMC2592349

[5] Lobbes H (2023) [Pure red cell aplasia: diagnosis, classification and treatment]. Rev Med Interne 44(1):19–26. 10.1016/j.revmed.2022.10.385 36336519 10.1016/j.revmed.2022.10.385

[6] Wu X, Cheng L, Liu X, Sun Y, Li B, He G, Li J (2022) Clinical characteristics and outcomes of 100 adult patients with pure red cell aplasia. Ann Hematol 101(7):1493–1498. 10.1007/s00277-022-04847-2 35460389 10.1007/s00277-022-04847-2

[7] Sawada K, Hirokawa M, Fujishima N, Teramura M, Bessho M, Dan K, Tsurumi H, Nakao S, Urabe A, Omine M, Ozawa K, Group PCS (2007) Long-term outcome of patients with acquired primary idiopathic pure red cell aplasia receiving cyclosporine A. A nationwide cohort study in Japan for the PRCA collaborative study group. Haematologica 92(8):1021–1028. 10.3324/haematol.11192 17640861 10.3324/haematol.11192

[8] Gurnari C, Maciejewski JP (2021) How i manage acquired pure red cell aplasia in adults. Blood 137(15):2001–2009. 10.1182/blood.2021010898 33657207 10.1182/blood.2021010898 PMC8057257

[9] Wu X, Wang S, Lu X, Shen W, Qiao C, Wu Y, Lu R, Wang S, Zhang J, Hong M, Zhu Y, Li J, He G (2018) Response to cyclosporine A and corticosteroids in adult patients with acquired pure red cell aplasia: serial experience at a single center. Int J Hematol 108(2):123–129. 10.1007/s12185-018-2446-y 29589280 10.1007/s12185-018-2446-y

[10] Lacy MQ, Kurtin PJ, Tefferi A (1996) Pure red cell aplasia: association with large granular lymphocyte leukemia and the prognostic value of cytogenetic abnormalities. Blood 87(7):3000–3006 8639922

[11] Dragani M, Andreani G, Familiari U, Marci V, Rege-Cambrin G (2020) Pure red cell aplasia and amegakaryocytic thrombocytopenia in thymoma: the uncharted territory. Clin Case Rep 8(4):598–601. 10.1002/ccr3.2642 32274018 10.1002/ccr3.2642 PMC7141733

[12] Sun L, Babushok DV (2020) Secondary myelodysplastic syndrome and leukemia in acquired aplastic anemia and paroxysmal nocturnal hemoglobinuria. Blood 136(1):36–49. 10.1182/blood.2019000940 32430502 10.1182/blood.2019000940 PMC7332901

[13] Fujishima N, Kohmaru J, Koyota S, Kuba K, Saga T, Omokawa A, Moritoki Y, Ueki S, Ishida F, Nakao S, Matsuda A, Ohta A, Tohyama K, Yamasaki H, Usuki K, Nakashima Y, Sato S, Miyazaki Y, Nannya Y, Ogawa S, Sawada K, Mitani K, Hirokawa M (2021) Clonal hematopoiesis in adult pure red cell aplasia. Sci Rep 11(1):2253. 10.1038/s41598-021-81890-5 33500526 10.1038/s41598-021-81890-5 PMC7838416

[14] Kwok B, Hall JM, Witte JS, Xu Y, Reddy P, Lin K, Flamholz R, Dabbas B, Yung A, Al-Hafidh J, Balmert E, Vaupel C, El Hader C, McGinniss MJ, Nahas SA, Kines J, Bejar R (2015) MDS-associated somatic mutations and clonal hematopoiesis are common in idiopathic cytopenias of undetermined significance. Blood 126(21):2355–2361. 10.1182/blood-2015-08-667063 26429975 10.1182/blood-2015-08-667063 PMC4653764

[15] Long Z, Li H, Du Y, Chen M, Zhuang J, Han B (2020) Gene mutation profile in patients with acquired pure red cell aplasia. Ann Hematol 99(8):1749–1754. 10.1007/s00277-020-04154-8 32594217 10.1007/s00277-020-04154-8

[16] Kawakami T, Sekiguchi N, Kobayashi J, Imi T, Matsuda K, Yamane T, Nishina S, Senoo Y, Sakai H, Ito T, Koizumi T, Hirokawa M, Nakao S, Nakazawa H, Ishida F (2018) Frequent STAT3 mutations in CD8(+) T cells from patients with pure red cell aplasia. Blood Adv 2(20):2704–2712. 10.1182/bloodadvances.2018022723 30337298 10.1182/bloodadvances.2018022723 PMC6199660

[17] Masuda M, Teramura M, Matsuda A, Bessho M, Shimamoto T, Ohyashiki K, Omine M, Motoji T, Mizoguchi H (2005) Clonal T cells of pure red-cell aplasia. Am J Hematol 79(4):332–333. 10.1002/ajh.20374 16044445 10.1002/ajh.20374

[18] Balasubramanian SK, Sadaps M, Thota S, Aly M, Przychodzen BP, Hirsch CM, Visconte V, Radivoyevitch T, Maciejewski JP (2018) Rational management approach to pure red cell aplasia. Haematologica 103(2):221–230. 10.3324/haematol.2017.175810 29217782 10.3324/haematol.2017.175810 PMC5792266

[19] Grimm J, Simnica D, Jakel N, Paschold L, Willscher E, Schulze S, Dierks C, Al-Ali HK, Binder M (2022) Azacitidine-induced reconstitution of the bone marrow T cell repertoire is associated with superior survival in AML patients. Blood Cancer J 12(1):19. 10.1038/s41408-022-00615-7 35091554 10.1038/s41408-022-00615-7 PMC8799690

[20] Kumar P, Henikoff S, Ng PC (2009) Predicting the effects of coding non-synonymous variants on protein function using the SIFT algorithm. Nat Protoc 4(7):1073–1081. 10.1038/nprot.2009.86 19561590 10.1038/nprot.2009.86

[21] Weeks LD, Niroula A, Neuberg D, Wong W, Lindsley RC, Luskin M, Berliner N, Stone RM, DeAngelo DJ, Soiffer R, Uddin MM, Griffin G, Vlasschaert C, Gibson CJ, Jaiswal S, Bick AG, Malcovati L, Natarajan P, Ebert BL (2023) Prediction of risk for myeloid malignancy in clonal hematopoiesis. NEJM Evid 2(5). 10.1056/evidoa2200310 10.1056/evidoa2200310 PMC10361696 37483562

[22] Thibile S, Barrett C, Potgieter S, Joubert G, Malherbe J (2022) Adult pure red cell aplasia at Universitas academic hospital, Bloemfontein, South africa: a 9-year review. S Afr Med J 112(9):753–759. 10.7196/SAMJ.2022.v112i9.16416 36214038 10.7196/SAMJ.2022.v112i9.16416

[23] Kawahara S, Sasaki M, Isobe Y, Ando J, Noguchi M, Koike M, Hirano T, Oshimi K, Sugimoto K (2009) Clinical analysis of 52 patients with granular lymphocyte proliferative disorder (GLPD) showed frequent anemia in indolent T-cell GLPD in Japan. Eur J Haematol 82(4):308–314. 10.1111/j.1600-0609.2009.01213.x 19220421 10.1111/j.1600-0609.2009.01213.x

[24] Kawakami T, Nakazawa H, Ishida F (2022) Somatic mutations in acquired pure red cell aplasia. Semin Hematol 59(3):131–136. 10.1053/j.seminhematol.2022.07.001 36115689 10.1053/j.seminhematol.2022.07.001

[25] Biancon G, Joshi P, Zimmer JT, Hunck T, Gao Y, Lessard MD, Courchaine E, Barentine AES, Machyna M, Botti V, Qin A, Gbyli R, Patel A, Song Y, Kiefer L, Viero G, Neuenkirchen N, Lin H, Bewersdorf J, Simon MD, Neugebauer KM, Tebaldi T, Halene S (2022) Precision analysis of mutant U2AF1 activity reveals deployment of stress granules in myeloid malignancies. Mol Cell 82(6):1107–1122e1107. 10.1016/j.molcel.2022.02.025 35303483 10.1016/j.molcel.2022.02.025 PMC8988922

[26] Smith MA, Choudhary GS, Pellagatti A, Choi K, Bolanos LC, Bhagat TD, Gordon-Mitchell S, Von Ahrens D, Pradhan K, Steeples V, Kim S, Steidl U, Walter M, Fraser IDC, Kulkarni A, Salomonis N, Komurov K, Boultwood J, Verma A, Starczynowski DT (2019) U2AF1 mutations induce oncogenic IRAK4 isoforms and activate innate immune pathways in myeloid malignancies. Nat Cell Biol 21(5):640–650. 10.1038/s41556-019-0314-5 31011167 10.1038/s41556-019-0314-5 PMC6679973

[27] Guglielmelli P, Calabresi L (2021) The MPL mutation. Int Rev Cell Mol Biol 365:163–178. 10.1016/bs.ircmb.2021.09.003 34756243 10.1016/bs.ircmb.2021.09.003

[28] Dhuri K, Alachkar H (2024) Differences in the mutational landscape of clonal hematopoiesis of indeterminate potential among races and between male and female patients with cancer. Exp Hematol 138:104271. 10.1016/j.exphem.2024.104271 38969020 10.1016/j.exphem.2024.104271 PMC12045072

[29] Peres LC, Colin-Leitzinger CM, Teng M, Dutil J, Alugubelli RR, DeAvila G, Teer JK, Du D, Mo Q, Siegel EM, Hampton OA, Alsina M, Brayer J, Blue B, Baz R, Silva AS, Nishihori T, Shain KH, Gillis N (2022) Racial and ethnic differences in clonal hematopoiesis, tumor markers, and outcomes of patients with multiple myeloma. Blood Adv 6(12):3767–3778. 10.1182/bloodadvances.2021006652 35500227 10.1182/bloodadvances.2021006652 PMC9631567

[30] Kawakami T, Kawakami F, Matsuzawa S, Yamane T, Mizuno Y, Asakura A, Higano D, Miyairi S, Sakai K, Nishina S, Sakai H, Kubota Y, Higuchi Y, Nakazawa H, Ishida F (2025) Mutational heterogeneities in STAT3 and clonal hematopoiesis-related genes in acquired pure red cell aplasia. Ann Hematol 104(3):1471–1479. 10.1007/s00277-025-06356-4 40202536 10.1007/s00277-025-06356-4 PMC12031804

[31] Yoshizato T, Dumitriu B, Hosokawa K, Makishima H, Yoshida K, Townsley D, Sato-Otsubo A, Sato Y, Liu D, Suzuki H, Wu CO, Shiraishi Y, Clemente MJ, Kataoka K, Shiozawa Y, Okuno Y, Chiba K, Tanaka H, Nagata Y, Katagiri T, Kon A, Sanada M, Scheinberg P, Miyano S, Maciejewski JP, Nakao S, Young NS, Ogawa S (2015) Somatic mutations and clonal hematopoiesis in aplastic anemia. N Engl J Med 373(1):35–47. 10.1056/NEJMoa1414799 26132940 10.1056/NEJMoa1414799 PMC7478337

[32] Genovese G, Kahler AK, Handsaker RE, Lindberg J, Rose SA, Bakhoum SF, Chambert K, Mick E, Neale BM, Fromer M, Purcell SM, Svantesson O, Landen M, Hoglund M, Lehmann S, Gabriel SB, Moran JL, Lander ES, Sullivan PF, Sklar P, Gronberg H, Hultman CM, McCarroll SA (2014) Clonal hematopoiesis and blood-cancer risk inferred from blood DNA sequence. N Engl J Med 371(26):2477–2487. 10.1056/NEJMoa1409405 25426838 10.1056/NEJMoa1409405 PMC4290021

[33] Jaiswal S, Fontanillas P, Flannick J, Manning A, Grauman PV, Mar BG, Lindsley RC, Mermel CH, Burtt N, Chavez A, Higgins JM, Moltchanov V, Kuo FC, Kluk MJ, Henderson B, Kinnunen L, Koistinen HA, Ladenvall C, Getz G, Correa A, Banahan BF, Gabriel S, Kathiresan S, Stringham HM, McCarthy MI, Boehnke M, Tuomilehto J, Haiman C, Groop L, Atzmon G, Wilson JG, Neuberg D, Altshuler D, Ebert BL (2014) Age-related clonal hematopoiesis associated with adverse outcomes. N Engl J Med 371(26):2488–2498. 10.1056/NEJMoa1408617 25426837 10.1056/NEJMoa1408617 PMC4306669

[34] Laurie CC, Laurie CA, Rice K, Doheny KF, Zelnick LR, McHugh CP, Ling H, Hetrick KN, Pugh EW, Amos C, Wei Q, Wang LE, Lee JE, Barnes KC, Hansel NN, Mathias R, Daley D, Beaty TH, Scott AF, Ruczinski I, Scharpf RB, Bierut LJ, Hartz SM, Landi MT, Freedman ND, Goldin LR, Ginsburg D, Li J, Desch KC, Strom SS, Blot WJ, Signorello LB, Ingles SA, Chanock SJ, Berndt SI, Le Marchand L, Henderson BE, Monroe KR, Heit JA, de Andrade M, Armasu SM, Regnier C, Lowe WL, Hayes MG, Marazita ML, Feingold E, Murray JC, Melbye M, Feenstra B, Kang JH, Wiggs JL, Jarvik GP, McDavid AN, Seshan VE, Mirel DB, Crenshaw A, Sharopova N, Wise A, Shen J, Crosslin DR, Levine DM, Zheng X, Udren JI, Bennett S, Nelson SC, Gogarten SM, Conomos MP, Heagerty P, Manolio T, Pasquale LR, Haiman CA, Caporaso N, Weir BS (2012) Detectable clonal mosaicism from birth to old age and its relationship to cancer. Nat Genet 44(6):642–650. 10.1038/ng.2271 22561516 10.1038/ng.2271 PMC3366033

[35] Xie M, Lu C, Wang J, McLellan MD, Johnson KJ, Wendl MC, McMichael JF, Schmidt HK, Yellapantula V, Miller CA, Ozenberger BA, Welch JS, Link DC, Walter MJ, Mardis ER, Dipersio JF, Chen F, Wilson RK, Ley TJ, Ding L (2014) Age-related mutations associated with clonal hematopoietic expansion and malignancies. Nat Med 20(12):1472–1478. 10.1038/nm.3733 25326804 10.1038/nm.3733 PMC4313872

[36] Jaiswal S, Ebert BL (2019) Clonal hematopoiesis in human aging and disease. Science. 10.1126/science.aan4673 31672865 10.1126/science.aan4673 PMC8050831

[37] Nazha A, Hu ZH, Wang T, Lindsley RC, Abdel-Azim H, Aljurf M, Bacher U, Bashey A, Cahn JY, Cerny J, Copelan E, DeFilipp Z, Diaz MA, Farhadfar N, Gadalla SM, Gale RP, George B, Gergis U, Grunwald MR, Hamilton B, Hashmi S, Hildebrandt GC, Inamoto Y, Kalaycio M, Kamble RT, Kharfan-Dabaja MA, Lazarus HM, Liesveld JL, Litzow MR, Majhail NS, Murthy HS, Nathan S, Nishihori T, Pawarode A, Rizzieri D, Sabloff M, Savani BN, Schachter L, Schouten HC, Seo S, Shah NN, Solh M, Valcarcel D, Vij R, Warlick E, Wirk B, Wood WA, Yared JA, Alyea E, Popat U, Sobecks RM, Scott BL, Nakamura R, Saber W (2020) A personalized prediction model for outcomes after allogeneic hematopoietic cell transplant in patients with myelodysplastic syndromes. Biol Blood Marrow Transplant 26(11):2139–2146. 10.1016/j.bbmt.2020.08.003 32781289 10.1016/j.bbmt.2020.08.003 PMC7609542

[38] Madan V, Cao Z, Teoh WW, Dakle P, Han L, Shyamsunder P, Jeitany M, Zhou S, Li J, Nordin HBM, Shi J, Yu S, Yang H, Hossain MZ, Chng WJ, Koeffler HP (2022) *Zrsr1* co-operates with *Zrsr2* in regulating splicing of U12-type introns in murine hematopoietic cells. Haematologica 107(3):680–689. 10.3324/haematol.2020.260562 33691379 10.3324/haematol.2020.260562 PMC8883539

[39] Lobbes H, Lega JC, Le Guenno G, Ruivard M, Mainbourg S (2023) Treatment strategy for acquired pure red cell aplasia: a systematic review and meta-analysis. Blood Adv 7(21):6451–6465. 10.1182/bloodadvances.2023010587 37624775 10.1182/bloodadvances.2023010587 PMC10632686

[40] Zhang RX, Huang YZ, Han B (2023) [Comparison of cyclosporine A and cyclosporine A combined with corticosteroid in the treatment of acquired pure red cell aplasia]. Zhongguo Shi Yan Xue Ye Xue Za Zhi 31(4):1138–1142. 10.19746/j.cnki.issn.1009-2137.2023.04.032 37551489 10.19746/j.cnki.issn.1009-2137.2023.04.032

[41] Rajala HL, Olson T, Clemente MJ, Lagstrom S, Ellonen P, Lundan T, Hamm DE, Zaman SA, Lopez Marti JM, Andersson EI, Jerez A, Porkka K, Maciejewski JP, Loughran TP, Mustjoki S (2015) The analysis of clonal diversity and therapy responses using STAT3 mutations as a molecular marker in large granular lymphocytic leukemia. Haematologica 100(1):91–99. 10.3324/haematol.2014.113142 25281507 10.3324/haematol.2014.113142 PMC4281318

