# Supplementary figures and images for: Comprehensive analysis of the clinical feature, myeloid neoplasm-related gene mutation profiles and T cell diversity acquired pure red cell aplasia
Source: Ann Hematol. 2025 Sep 25;104(9):4411–27. doi: 10.1007/s00277-025-06638-x (PMC12552327; doi:10.1007/s00277-025-06638-x)

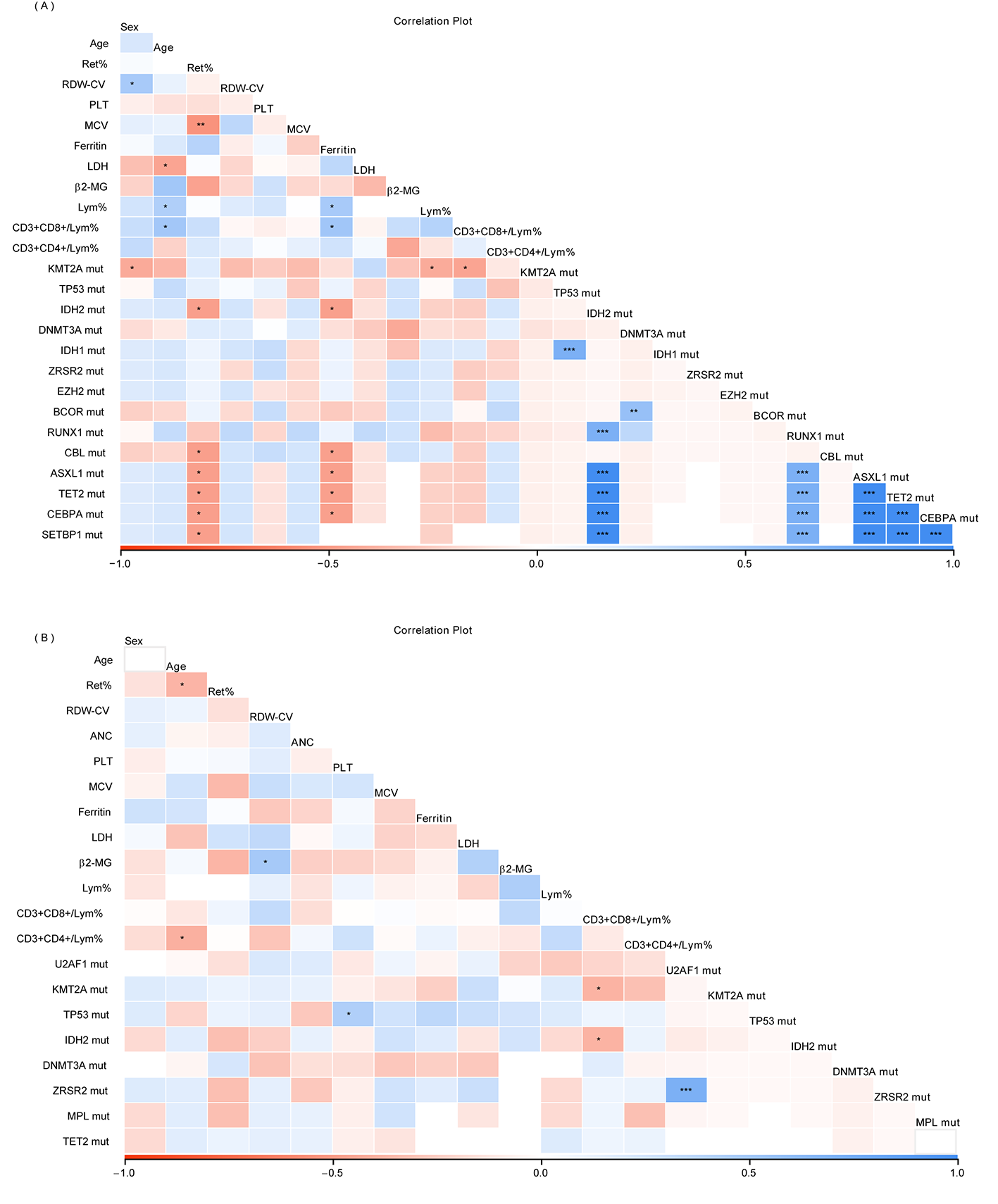

Supplement: Supplementary file 1 — Correlation of clinical information with mutation and clonal diversity in primary PRCA patients and LGLL-PRCA patients. (A) Correlation plot of clinical information with mutation in primary PRCA patient; (B) Correlation plot of clinical information with mutation in LGLL-PRCA patient. (PNG 320 KB) [file 277_2025_6638_Fig6_ESM.png]

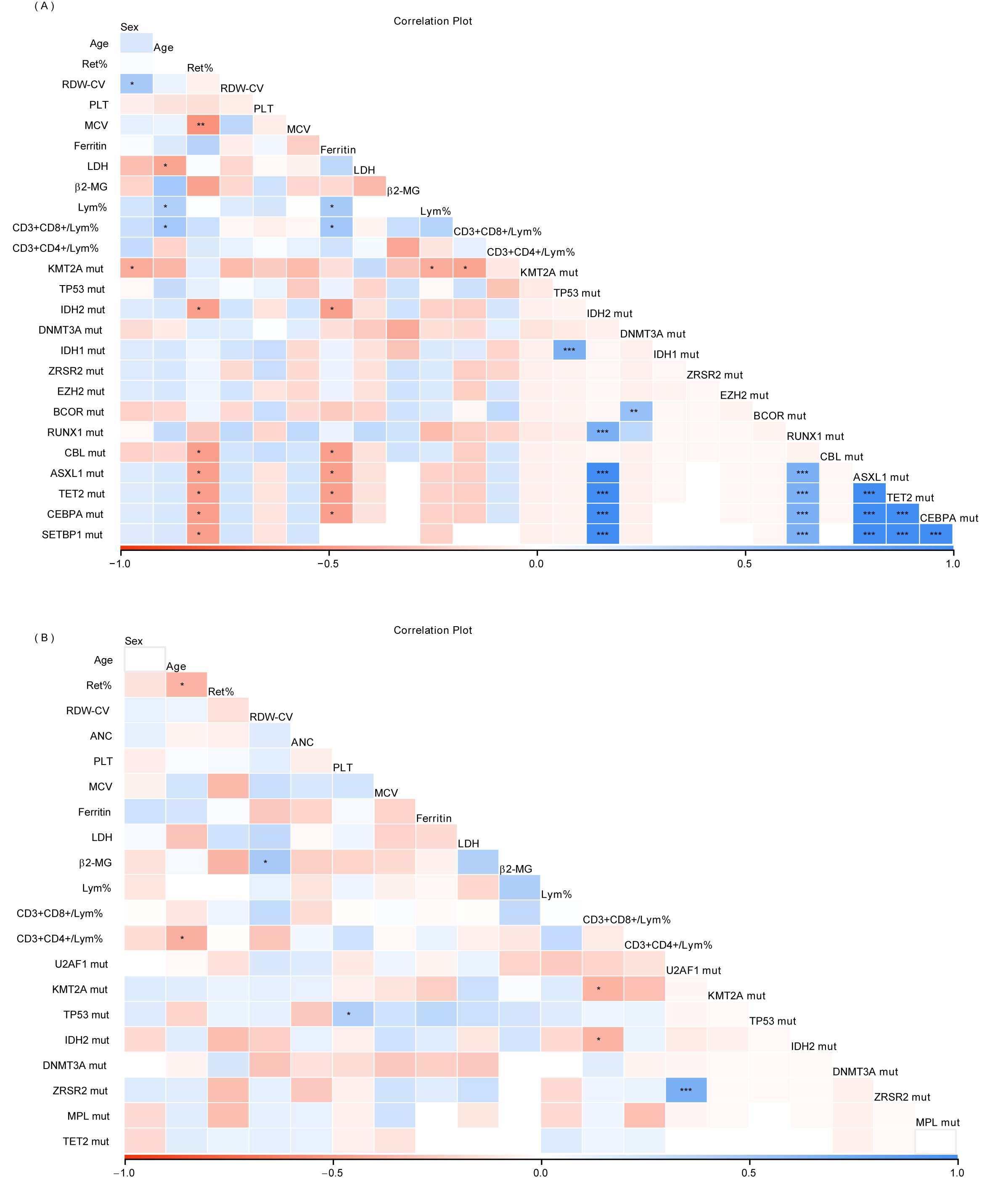

Supplement: Supplementary file 2 — High Resolution Image (TIF 1.63 MB) [file 277_2025_6638_MOESM1_ESM.tif]

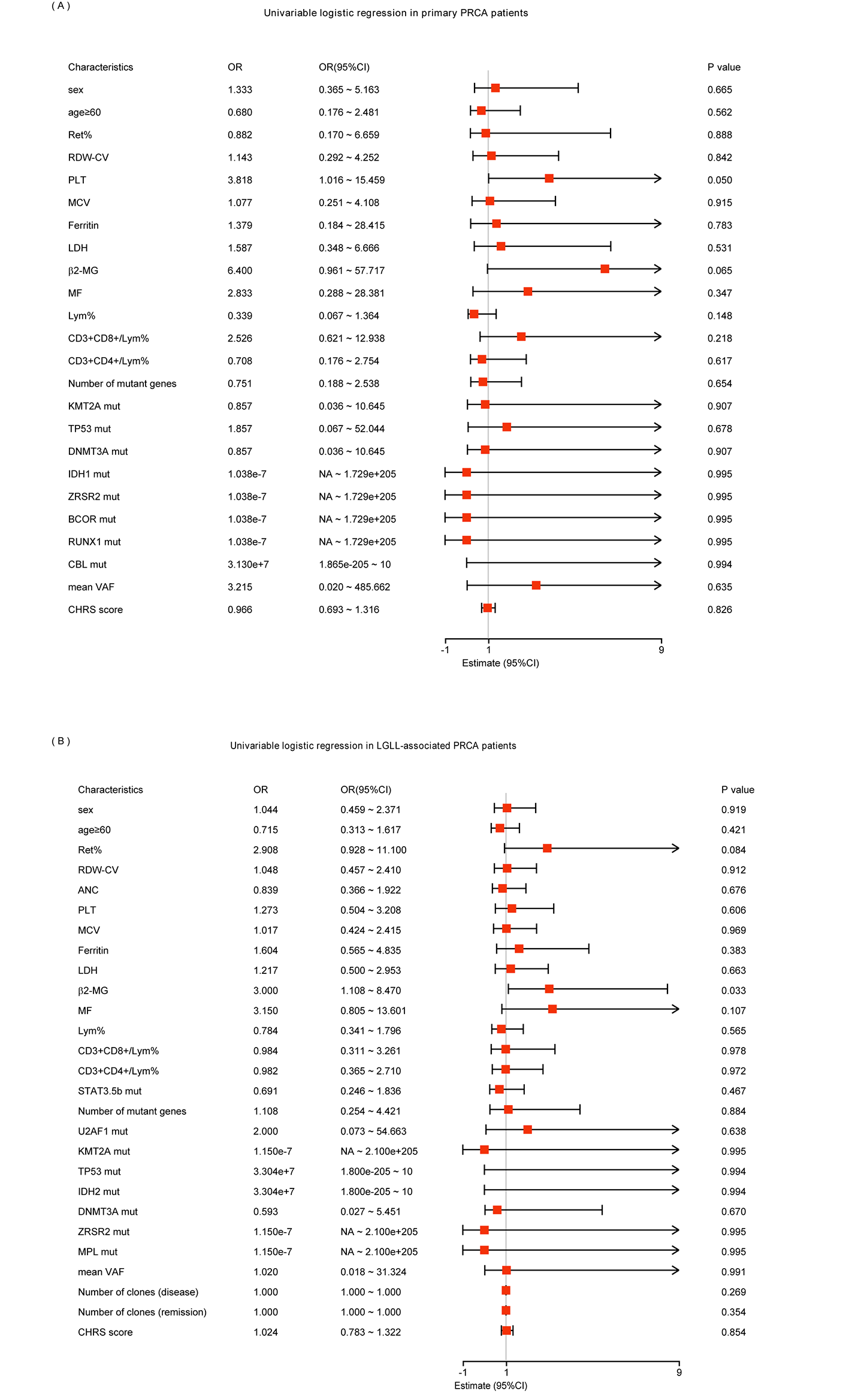

Supplement: Supplementary file 3 — Univariable logistic regression in primary PRCA and LGLL-PRCA patients. (A) Univariable logistic regression in primary PRCA patients; (B) Univariable logistic regression in LGLL-PRCA patients. (PNG 205 KB) [file 277_2025_6638_Fig7_ESM.png]

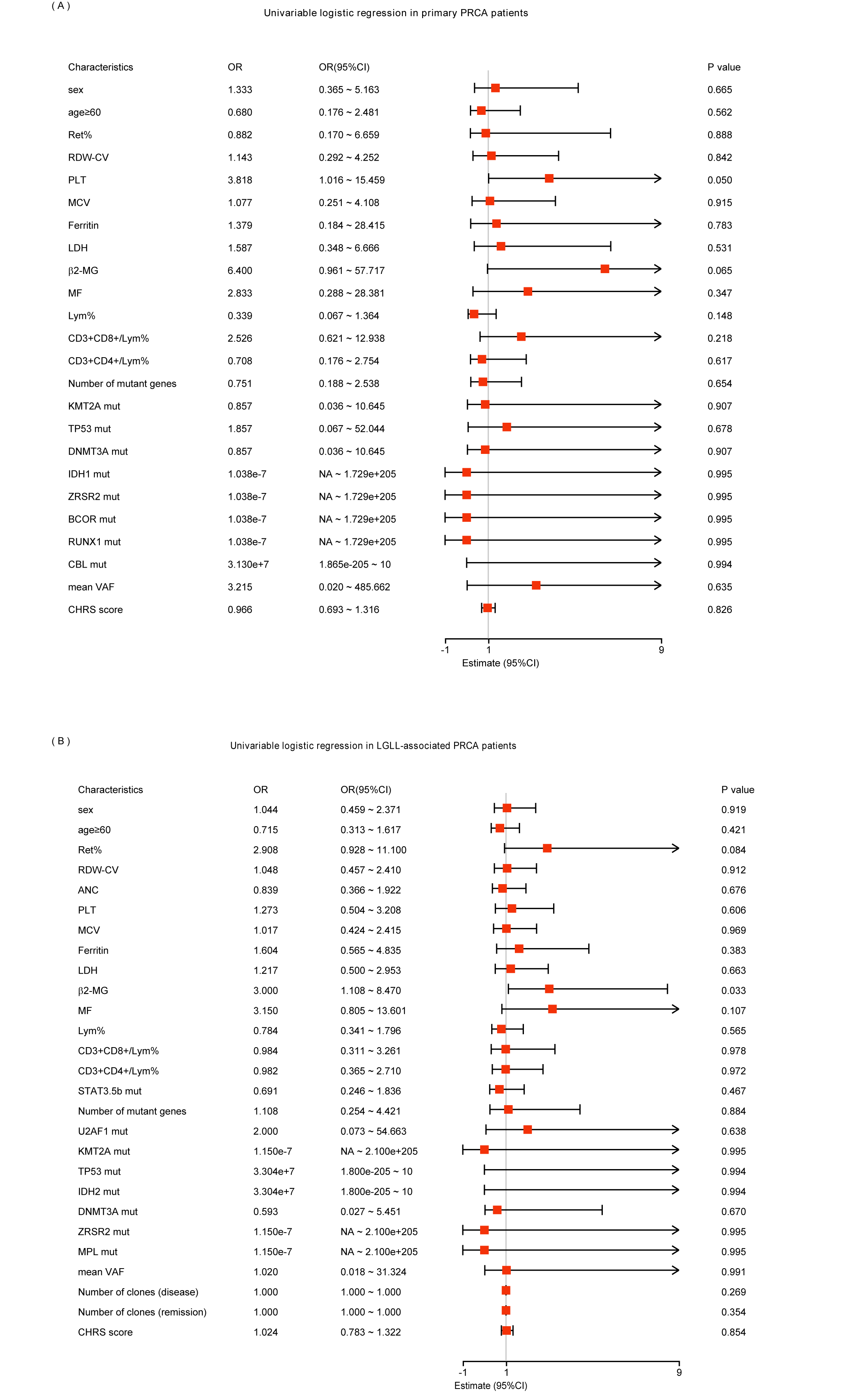

Supplement: Supplementary file 4 — High Resolution Image (TIF 1.76 MB) [file 277_2025_6638_MOESM2_ESM.tif]
